# Variation in the Microbiota Associated with *Daphnia magna* Across Genotypes, Populations, and Temperature

**DOI:** 10.1007/s00248-019-01412-9

**Published:** 2019-08-03

**Authors:** Jonas Frankel-Bricker, Michael J. Song, Maia J. Benner, Sarah Schaack

**Affiliations:** 1 Department of Biology, Reed College, Portland, OR 97202 USA; 2 Present Address: Department of Biological Sciences, Boise State University, Boise, ID 83725 USA; 3 University and Jepson Herbaria and Department of Integrative Biology, University of California, Berkeley, CA 94720 USA

**Keywords:** Microbiome, Cladocera, Water fleas, Climate change, Reproducibility

## Abstract

Studies of how the microbiome varies among individuals, populations, and abiotic conditions are critical for understanding this key component of an organism’s biology and ecology. In the case of *Daphnia*, aquatic microcrustaceans widely used in population/community ecology and environmental science studies, understanding factors that influence microbiome shifts among individuals is useful for both basic and applied research contexts. In this study, we assess differences in the microbiome among genotypes of *D. magna* collected from three regions along a large latitudinal gradient (Finland, Germany, and Israel). After being reared in the lab for many years, we sought to characterize any differences in genotype- or population-specific microbial communities, and to assess whether the microbiota varied among temperatures. Our study is similar to a recent comparison of the microbial communities among *D. magna* genotypes raised in different temperatures published by Sullam et al. (Microb Ecol 76(2):506-517, 2017), and as such represents one of the first examples of a reproducible result in microbiome research. Like the previous study, we find evidence for a strong effect of temperature on the microbiome of *D. magna*, although across a much smaller temperature range representing potential near-future climates. In addition, we find evidence that the microbiomes of *D. magna* genotypes from different regions are distinct, even years after being brought into the laboratory. Finally, our results highlight a potentially common finding in the expanding area of microbiome research—differences among treatments are not necessarily observed in the most abundant taxonomic groups. This highlights the importance of considering sampling scheme and depth of coverage when characterizing the microbiome, as different experimental designs can significantly impact taxon-specific results, even when large-scale effects are reproduced.

**Electronic supplementary material:**

The online version of this article (10.1007/s00248-019-01412-9) contains supplementary material, which is available to authorized users.

## Introduction

There is growing interest in understanding variation in the microbial communities associated with organisms, including the degree to which such communities vary among individuals, species, and environments. The microbial community associated with a host organism, referred to as its microbiome, is composed of facultative and obligate symbiotic bacteria, fungi, protists, and other microorganisms. Metagenomic studies have utilized next-generation sequencing methods and bioinformatic workflows to survey these communities in a wide array of study systems. Beyond initial surveys, more recent laboratory and field experiments have been performed to uncover the effects of abiotic factors on microbial community composition and structure to investigate main effects, fluctuating interactions, and microbiome plasticity (e.g., [20]; reviewed in [42]). Importantly, studies looking at factors affecting microbiota can fall into one of several categories: carry-over studies (which can be conducted in an axenic or xenic laboratory environment), colonization studies (where organisms are made germ-free and inoculated with uniform microbiota to assess which of a common set of microbes persist), or natural variation studies (where microbiota from recently collected animals are compared). Environmental factors such as pH, salinity, and diet can lead to variation in microbial communities across environmental gradients [8, 11, 15, 28, 37, 43]. Temperature, in particular, has also been examined, in part due to interest in understanding the effects of global climate change on microbial communities and host-microbe interactions [12, 17, 24, 30]. In addition, studies have explored the relationship between functional aspects of the microbiome and important host traits, such as disease resistance and fitness (e.g., in mosquitoes [10, 19], *Drosophila* [9, 16], and *Caenorhabditis elegans* [3]).

**Fig. 1:**
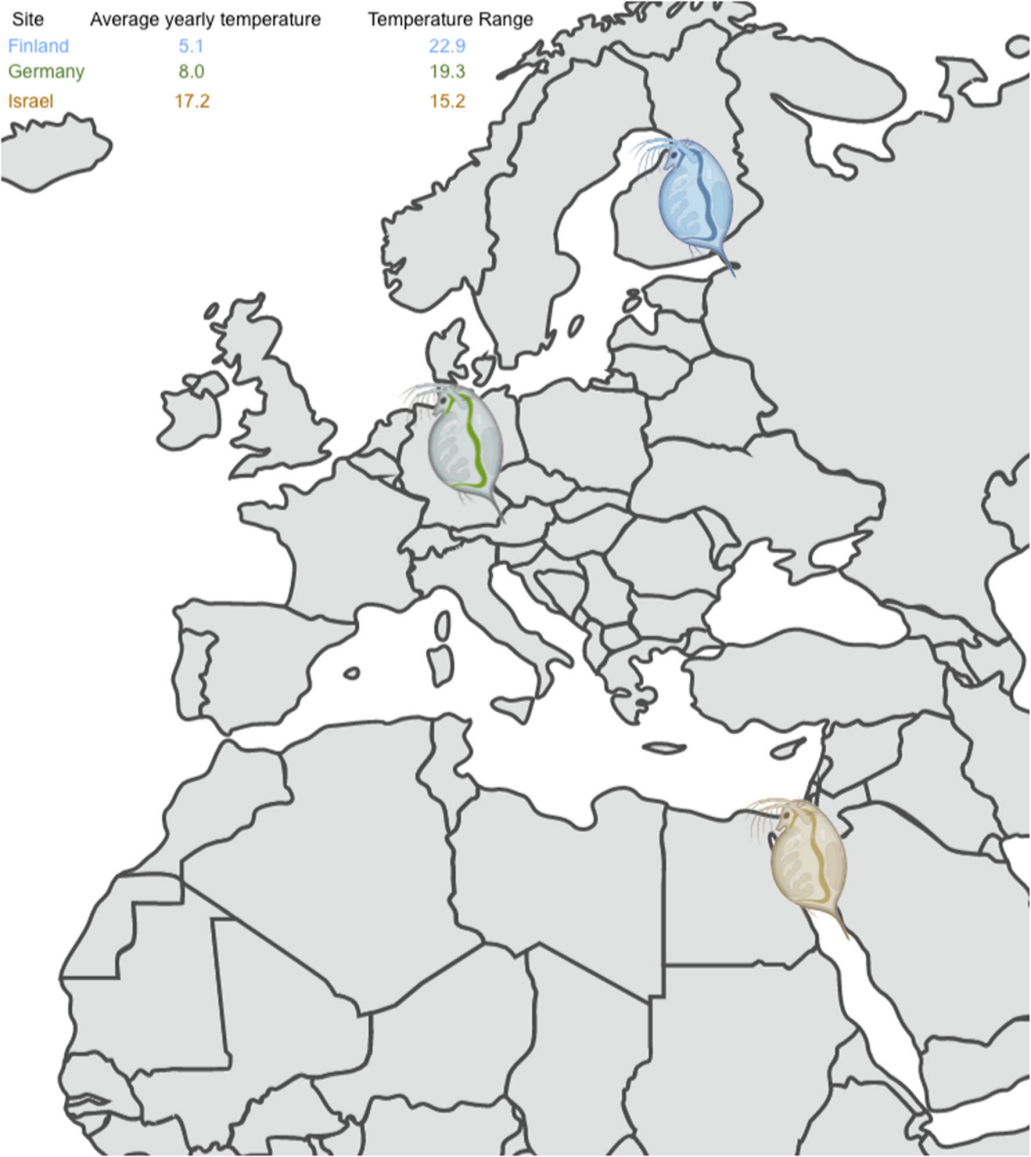
Map of collection sites in Finland (blue), Germany (green), and Israel (yellow) for the nine genotypes used in this study including data on temperature (Celsius; see also Table S1)

Understanding the feedbacks between hosts and their associated microbiomes and investigating whether environmental conditions affect microbe-host interactions is now widely considered to be an important area of inquiry in ecology and evolution. The planktonic crustacean *Daphnia magna* (Cladocera) is an important model organism in population and community ecology, evolution, ecotoxicology, and genomics [35]. They also serve as a bioindicator species for ecosystem health and, thus, are an important focus for research on the ecological impacts of global climate change. Research has shown that, as with other study systems, changes to microbial communities affect *Daphnia* fitness [5, 6, 26, 40, 41]. There is evidence from other systems that host genotype and environmental conditions may have an interactive effect on host microbiomes [1, 7], and a recent study in *D. magna* provided the first report of such dynamics in this species [44]. Here, we perform a carry-over study in an axenic laboratory environment to examine the microbiome of three different genotypes from three populations of *D. magna* originally collected along a latitudinal gradient with distinct ecological and climatic conditions (Fig. 1). Further, we test if *D. magna* microbiomes from these nine genotypes vary when raised in different temperatures under laboratory conditions. We hypothesized that population-of-origin and temperature (and, potentially, their interaction) would exhibit an effect on microbiota given that bacteria have been shown to vary across temperatures in other systems ([2, 32]), and the history of different temperatures experienced by the isolates we tested may have shaped their capacity for hosting different bacteria. We compare our results to those of Sullam et al. [44], who also examined whether population (“host clonal line”) or temperature affects the microbiome, although several aspects of their study design differed from ours. The overall goal of our study is to assess whether variation in the microbiome is associated with genotype-/population-level specificity, temperature-dependent effects, or the interaction of the two. We discuss the implications of our findings for understanding potential effects of a changing climate, comparative metagenomics, and the reproducibility of independently-performed microbiome studies.

## Methods

### Experimental Design

*Daphnia magna* lines used in this experiment were clonal descendants of lineages originally collected and later hatched in the laboratory between 2000 and 2008 which originated from three locales (“Population”): Finland (F), Israel (I), and Germany (G) (see Fig. 1 and Supplemental Table S1). Three genotypes were assayed from each location, for a total of 9 genotypes: (Finland [FA, FB, FC], Israel [IA, IB, IC], and Germany [GA, GB, GC]), with two replicates of each genotype (“SourceRep”; referred to as C1 and C2) maintained independently. Genotypes from Israel and Finland used in this study were collected from the same locations as Israel and Finnish clones used in Sullam et al. [44], but were not the same genotypes used in that study.

Prior to the experiment, animals were reared in Aachener Daphnien Medium (ADaM; [22]) in 3.7-l jars (“Jar”) in growth chambers at 18 °C in a 16:8 light/dark cycle. Animals were transferred and acclimated to experimental temperatures (Low [16 °C], Medium [18 °C], and High [20 °C]) for a minimum of 2 weeks prior to the start of the experiment. *Daphnia* were fed an axenic culture of *Scenedesmus obliquus* (UTEX #393) cultivated at room temperature in autoclaved Bold’s Basal Medium (BBM). New cultures were seeded weekly and presumed to be axenic and thus not a substantial source of new microbes in the experiment, however cultures were not sequenced nor analyzed for contamination.

To initiate the treatment period, four juvenile females from each replicate genotype were raised at each temperature (a total of 8 experimental animals per genotype per temperature) by transferring individuals to separate sterilized 200-ml glass beakers containing 150 ml of autoclaved ADaM. Beakers were covered with 4 layers of autoclaved Miracloth (cat. no. 475855) during the experimental period to mitigate airborne contamination. Animals were maintained at each of the experimental temperatures for three generations (~ 6 weeks). *Daphnia* were transferred to new beakers using sterile technique every 3 days (before each feeding), and all animal handling was performed in a fume hood next to a Bunsen burner. *Daphnia* were fed 5–10 ml of *S. obliquus* suspended in autoclaved ADaM at a concentration of 95–97% transmittance at 680 nm, which corresponds to approximately 600,000 cells/ml. In each experimental beaker, progeny from the first clutch were discarded. Animals from the second clutch were allowed to mature for 2–3 days to allow for sex identification. A single juvenile female was then selected and transferred to a new beaker to propagate the experimental line. Offspring from the second clutch of the third generation were reared together, and a single large juvenile female (immediately preceding egg formation) was collected for DNA extraction (approximately 1–2 weeks after birth). If an experimental line went extinct prior to harvesting tissue, a new line was initiated by collecting a juvenile female from the source population jar and reared using the same protocol. Due to differential survivorship and development times, *Daphnia* were harvested for DNA extraction at many time points between September and December 2016 (see Supplemental Table S2 for final sample sizes after sequencing and sample metadata). While sample processing was not explicitly randomized, there was no association between extraction date and specific populations or genotypes.

In addition, microbial DNA was extracted from large juvenile females harvested from the 3.7-L jars of animals from which the experimental animals were drawn, both prior to (“pre-”) and after (“post-”) the experiment was conducted. These animals were not maintained using sterile technique, but were assayed to assess whether the standing variation in microbiota of lab-reared *Daphnia* varied over the course of the experiment (approximately 6 months). All but one pre-experiment sample were harvested between July 15 and July 28, 2016, and post-experimental samples were harvested between January 2 and January 23, 2017 (see Supplemental Table S2).

### Microbial DNA Extraction

Microbial DNA was extracted from live animals using the Zymo Research Fungal/Bacterial DNA Microprep kit (#D6007) following the manufacturer’s protocol with modifications. *Daphnia* were individually collected in sterile 1.5 ml microcentrifuge tubes. Lysis buffer (250 μl) was added to the tube, and whole *Daphnia* were manually disrupted for 1–2 min using a sterilized pestle. Additional lysis buffer (500 μl) was added to the tube, and the tube contents were transferred to bead tubes provided by the kit. Bead tubes were disrupted using a table-top vortex on the maximum setting for 5 min. Spin filters were allowed to air dry for 1 min to evaporate excess wash buffer prior to the elution step. DNA elution buffer was heated to 45 °C and allowed to soak onto the spin filter for 3–5 min prior to the final elution step. Two blank DNA extractions were carried out to test for contamination of extraction kits. Another negative control extraction was performed on autoclaved ADaM to test for laboratory contamination of the growth media. Two PCR reactions were prepared without DNA template and sequenced to test for contamination during sequencing library preparation. Microbial DNA extracted from each sample was quantified using a Qubit Fluorometer and stored at − 80 °C and, in all cases, the bacterial content in the negative controls was below detectable levels.

### Amplification and Sequencing of Microbial 16S rDNA

The V3-V4 hypervariable region of microbial 16S rDNA was amplified using the primer pair 341F-785R [21] using linker sequences [45] and CS Tags (adapter sequences) provided by the University of Idaho Genomics Resources Core (Supplemental Table S3). Primary polymerase chain reactions (PCR 1) were performed using QuantaBio 5PRIME HotMasterMix following the manufacturer’s protocol. Reactions (50 μl) were prepared with 20 μl HotMasterMix, 26 μl Nuclease Free H_2_0, 1 μl F primer (10 μM), 1 μl R primer (10 μM), and 2 μl of template microbial DNA. Reactions were initiated in a thermocycler at 94 °C for 3 min to denature the DNA. Amplification steps were carried out at 94 °C for 45 s, 60 °C for 60 s, and 72 °C for 90 s, and repeated for 35 cycles, with a final extension step at 72 °C for 10 min. PCR products were visualized on 2% agarose gels. Secondary polymerase chain reactions (PCR 2) to attach linker, adaptor, and barcode sequences to the PCR 1 products were performed using the same kit. Reactions (25 μl) were prepared with 10 μl HotMasterMix, 12.8 μl Nuclease Free H_2_0, 0.9 μl Barcoded Primer (2 μM), and 1.25 μl of the PCR1 product as template. Reactions were initiated in a thermocycler at 94 °C for 90 s. Amplification steps were carried out at 94 °C for 30 s, 60 °C for 30 s, and 72 °C for 90 s, and repeated for 10 cycles, with a final extension step at 72 °C for 5 min. Secondary PCR products were quantified using a Qubit fluorometer and subsequently pooled and sequenced on the Illumina MiSeq V3 platform to generate 250 bp paired-end reads (see Supplemental Table S2 for raw reads produced and subsequent filtering steps).

### Data Analysis

Forward and reverse paired-end reads were filtered and trimmed to 250 and 240 bp, respectively (filtering parameters: no Ns allowed, maximum expected error [EE] score = 6 or less, and a FASTQ quality score [truncQ] cutoff of 2) using the DADA2 pipeline [4]. The DADA2 pipeline was used with default parameters to dereplicate and merge paired-end reads and remove chimeras. DADA2 generates amplicon sequence variants (ASVs) that are analogous to and an improvement on operational taxonomic units (OTUs) and we will be referring to the output as OTUs throughout the paper. Taxonomy was assigned to these OTUs using the DADA2-formatted reference SILVA database (Version 132, https://benjjneb.github.io/dada2/training.html; [33]).

To calculate phylogenetic distance, a neighbor joining (NJ) tree was inferred using the *phangorn* package in R [36] and a GTR+G+I maximum likelihood (ML) tree was inferred using the NJ tree as the starting point. The ML tree, the assigned OTUs, the read count data, and contextual sample metadata were combined in a *phyloseq* object for downstream analyses [27].

Data were rarified to 15,000 reads per sample and a permutational analysis of variance (PERMANOVA) was performed using the *adonis* function in the *vegan* package (v2.5-2; [14]) in R with 999 permutations to test whether temperature, population-of-origin, genotype, or their interaction had an effect on beta diversity measures. All statistical analyses were performed on weighted and unweighted UniFrac distances and Bray-Curtis dissimilarity (see Supplemental Table S4). Unweighted UniFrac distance compares microbiome compositions, but not relative abundance, across samples and accounts for phylogenetic distances among taxa. Both weighted UniFrac distance and Bray-Curtis dissimilarity compare microbiome compositions and relative abundances, however, only weighted UniFrac distance accounts for the phylogenetic distance among taxa. Results from PERMANOVA are sensitive to within-group variation, and homogeneity of group dispersion tests were performed for all factors identified to have a significant effect. Alpha diversity (Supplemental Table S5) was assessed using observed richness and Shannon and Simpson diversity indices across different temperatures, populations-of-origin, and genotype in *phyloseq*. ANOVAs were performed on the four most abundant families to estimate the effects of temperature, population-of-origin, and genotype on the relative abundance of taxa. On a non-rarified dataset, we used *DESeq2* [25] as well as a  random forest classifier [23] with default parameters  implemented in* microbiomeseq *(https://github.com/umerijaz/microbiomeSeq) to identify families that significantly differed between treatments and used phyloseq and microbiomeseq for various data visualizations (Supplemental Tables S6 and S7).

## Results

We sequenced a total of 101 experimental samples (9 from Finland, 48 from Germany, and 44 from Israel) and 103 pre- and post-experiment samples (see Supplemental Table S2 for detailed sample metadata). We generated an average of approximately 35,000 reads per sample and, following quality filtering and the removal of chimeras, approximately 25,000 reads per sample remained for downstream analysis (see Supplemental Table S2). Across 210 samples sequenced, we identified 1175 OTUs across 6 taxonomic ranks.

**Fig. 2:**
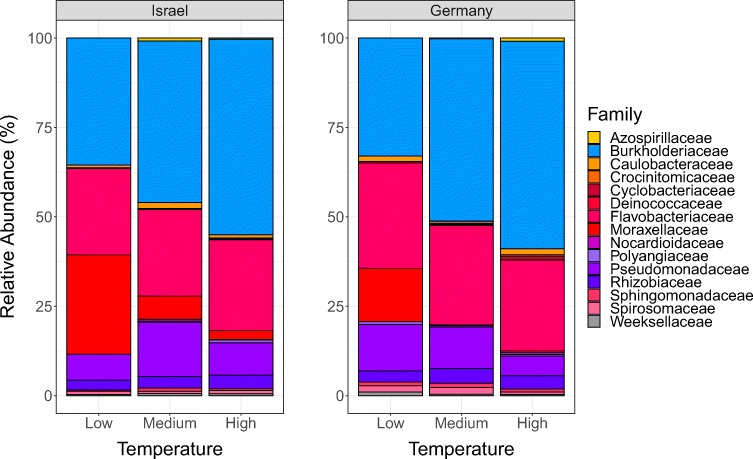
Average relative abundance of the top 15 families shared between pooled experimental samples from two populations Israel (left) and Germany (right) across three temperatures (Low = 16 °C, Medium = 18 °C, and High = 20 °C)

**Fig. 3:**
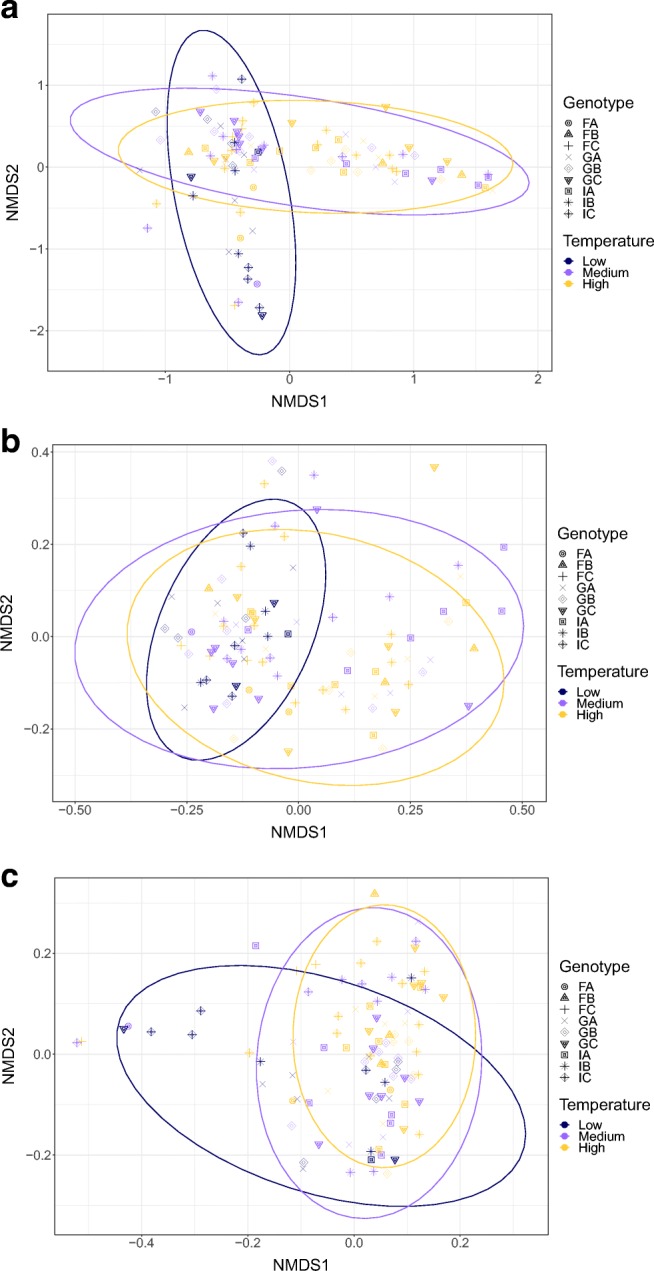
Non-metric multidimensional scaling (NMDS) plots based on **a** Bray-Curtis dissimilarity, **b** unweighted Unifrac distance, and **c** weighted UniFrac distance. Experimental samples are colored by temperature fitted with normal confidence ellipses, while shapes represent genotype

**Fig. 4:**
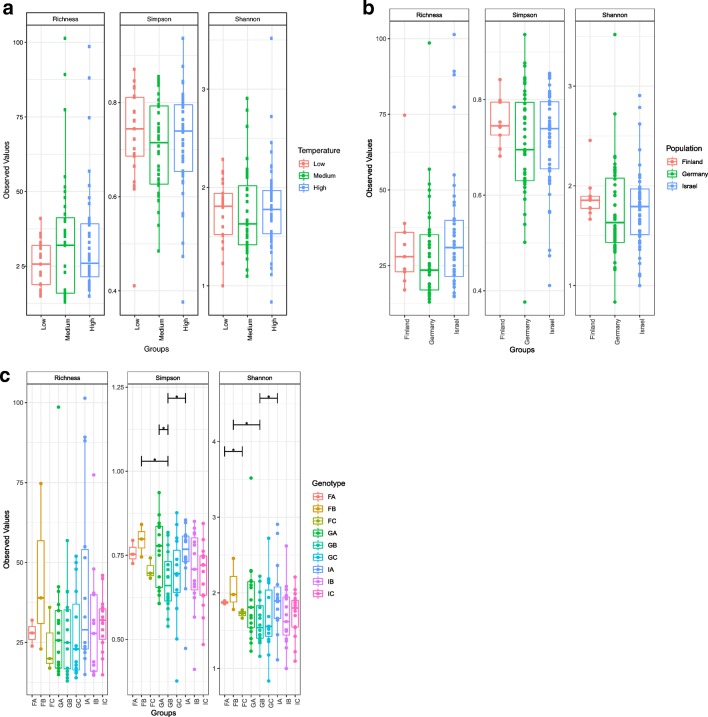
Simpson, richness, and Shannon indices of alpha diversity grouped by **a** temperature, **b** population-of-origin, and **c** genotype for experimental samples. Pairwise analysis of variance in diversity was performed between groups and significance below the *p* value threshold of 0.05 is indicated by asterisks

The primary factor influencing beta diversity measures in the microbiome was temperature, regardless of the metric used (see Table 1; Bray-Curtis dissimilarity [*df* = 2, *F* = 5.79, *p* = 0.001], unweighted UniFrac distance [*df* = 2, *F* = 5.11, *p* = 0.001], and weighted UniFrac distance [*df* = 2, *F* = 6.63, *p* = 0.001]), indicating that temperature influences both microbiome composition and structure. Data for the temperature effect using unweighted UniFrac distance failed the homogeneity of group dispersion test (*df* = 2, *F* = 7.11, *p* = 0.001), suggesting the differences detected may result from heteroscedasticity of the data. However, the effect of temperature is illustrated by post hoc pairwise comparisons (Table 2; Supplemental Table S4). Figure 2 shows the shift in the average relative abundance of just the top 15 families shared across pooled samples from (A) Israel and (B) Germany (samples from Finland were excluded from this analysis because of low survivorship leading to small sample sizes). The non-metric multidimensional scaling (NMDS) plots of all three metrics used show low-temperature treatments separate from medium- and high-temperature treatments (Fig. 3a–c). Overall, levels of alpha diversity within samples were not affected by temperature (Fig. 4a) and did not differ by population (Fig. 4b), although there were a few genotypes with relatively high and low levels of diversity (Fig. 4c) based on both Simpson and Shannon indices.

**Table 1:** Overall PERMANOVA results using measures of weighted UniFrac distance, unweighted UniFrac distance, and Bray-Curtis dissimilarity to test for main and interaction effects (see Supplemental Table S4 for all ANOVA tables)

	Degrees of freedom	Sum of squares	Mean square	F	*p*
Weighted UniFrac distance					
Population	2	0.193	0.097	4.883	*0.001*
Temperature	2	0.262	0.131	6.629	*0.001*
Extraction date	26	1.498	0.058	2.914	*0.001*
Genotype (nested in population)	6	0.208	0.035	1.752	0.032
Jar (nested in genotype and population)	7	0.200	0.029	1.447	0.107
Population × temperature	3	0.026	0.009	0.441	0.943
Genotype × temperature	8	0.138	0.017	0.874	0.624
Jar × temperature	9	0.184	0.020	1.036	0.449
Residuals	36	0.712	0.020		
Unweighted UniFrac distance					
Population	2	0.220	0.110	1.101	0.295
Temperature	2	1.019	0.510	5.108	*0.001*
Extraction date	26	6.227	0.239	2.401	*0.001*
Genotype (nested in population)	6	0.496	0.083	0.828	0.814
Jar (nested in genotype and population)	7	0.585	0.084	0.837	0.83
Population × temperature	3	0.245	0.082	0.819	0.738
Genotype × temperature	8	0.587	0.073	0.736	0.964
Jar × temperature	9	0.953	0.106	1.061	0.352
Residuals	36	3.591	0.100		
Bray-Curtis dissimilarity					
Population	2	0.687	0.344	3.028	*0.003*
Temperature	2	1.313	0.657	5.787	*0.001*
Extraction date	26	7.963	0.306	2.699	*0.001*
Genotype (nested in population)	6	0.902	0.150	1.324	0.108
Jar (nested in genotype and population)	7	1.205	0.172	1.517	0.033
Population × temperature	3	0.301	0.100	0.886	0.59
Genotype × temperature	8	0.999	0.125	1.100	0.312
Jar × temperature	9	1.369	0.152	1.341	0.08
Residuals	36	4.085	0.113	0.217	

Significant *p* values after Bonferroni correction are in italics

**Table 2:** Pairwise-PERMANOVA using measures of weighted UniFrac distance, unweighted UniFrac distance, and Bray-Curtis dissimilarity across temperature treatments (Low [16 °C], Medium [18 °C], and High [20 °C])

	Degrees of freedom	Sum of squares	*F*	*p*
Weighted UniFrac distance				
Medium vs high	1	0.063	1.824	0.123
Medium vs low	1	0.099	2.232	0.106
High vs low	1	0.223	6.136	*0.001*
Unweighted UniFrac distance				
Medium vs high	1	0.190	1.319	0.194
Medium vs low	1	0.518	3.843	*0.002*
High vs low	1	0.781	6.086	*0.001*
Bray-Curtis dissimilarity				
Medium vs high	1	0.256	1.486	0.151
Medium vs low	1	0.648	3.459	*0.009*
High vs low	1	0.899	4.778	*0.001*

Significant *p* values after Bonferroni correction are in italics

Population-of-origin had a significant, but smaller, effect than temperature on Bray-Curtis dissimilarity (*df* = 2, *F* = 3.03, *p* = 0.003) and weighted UniFrac distance (*df* = 2, *F* = 4.88, *p* = 0.001), while no effect was detected for unweighted UniFrac distance (*df* = 2, *F* = 1.10, *p* = 0.295), indicating that *Daphnia* from different geographic regions harbor microbial communities that differ in structure, but not composition (Table 1; Fig. 3). When looking at the most abundant families across samples (Burkholderiaceae, Flavobacteriaceae, Pseudomonadaceae, and Rhizobiaceae; Table 3 and Supplemental Table S6), the only significant factor is population-of-origin and it is only detectable for one of these prominent groups (Flavobacteriaceae; Table 3). Three of these families were also highly abundant in the microbiota extracted from *D. magna* by Sullam et al. [44], although *Limnohabitans* is classified within Burkholderiaceae in our study (using the SILVA database) but is included in Comamonadaceae in the GreenGenes database ([10.1038/ismej.2011.139] [13] used in their study.

**Table 3:** Temperature, population, and genotype effects for the top 4 most relatively abundant families (Burkholderiaceae, Flavobacteriaceae, Pseudomonadaceae, Rhizobiaceae) observed across all experimental samples at > 1% abundance

	Degrees of freedom	Sum of squares	Mean square	*F*	*p*
Burkholderiaceae					
Temperature	2	4.82	2.409	4.336	0.016
Population	2	3.26	1.630	2.934	0.059
Temperature × population	3	1.09	0.365	0.656	0.581
Temperature × genotype (nested in population)	14	5.68	0.405	0.730	0.7381
Residuals	79	43.89	0.556		
Flavobacteriaceae					
Temperature	2	0.159	0.080	0.247	0.782
Population	2	4.746	2.373	7.367	*0.001*
Temperature × population	3	0.934	0.311	0.967	0.413
Temperature × genotype (nested in population)	14	11.716	0.837	2.598	0.004
Residuals	77	24.804	0.322		
Pseudomonadaceae					
Temperature	2	4.24	2.119	4.310	0.017
Population	2	1.17	0.587	1.194	0.309
Temperature × population	3	4.04	1.346	2.738	0.049
Temperature × genotype (nested in population)	14	9.16	0.654	1.330	0.210
Residuals	77	37.86			
Rhizobiaceae					
Temperature	2	0.327	0.164	0.317	0.729
Population	2	1.003	0.502	0.974	0.384
Temperature × population	2	0.922	0.461	0.895	0.414
Temperature × genotype (nested in population)	14	13.768	0.983	1.909	0.044
Residuals	58	29.883	0.515		

Significant *p* values after Bonferroni correction are in bold

**Fig. 5:**
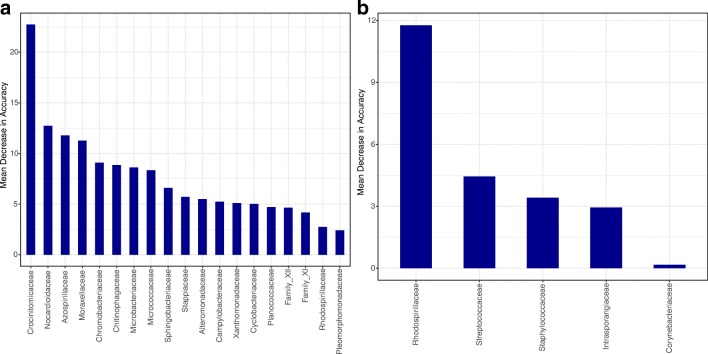
Mean decrease in accuracy of classification of experimental samples by **a** temperature treatment and **b** population-of-origin based on removal of each family using a random forest classifier. The families with the highest mean decrease in accuracy are considered the most important feature of the differential change in community composition across treatments (see Supplemental Table S8 for data)

**Fig. 6:**
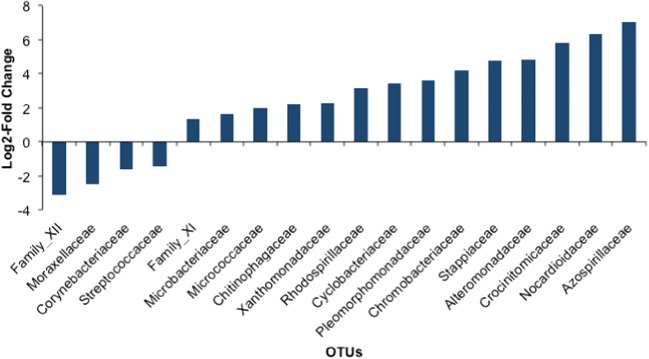
Log2-fold change in abundance based on temperature treatment for families based on pairwise comparisons between families observed in low- and high-temperature treatments (see Supplemental Table S7 for all significant log2-fold changes between low and medium and medium and high treatments). Negative values indicate higher abundance in the low-temperature treatment relative to the high-temperature treatment, and positive values indicate an increase in abundance of a particular family in high-temperature samples relative to low temperature

We used a random forest classifier (Liaw and Wiener 2002) in order to determine the relative importance of various taxa for community composition across levels for a given factor. The taxa that cause the highest mean decrease in accuracy when excluded are considered the most important for explaining a treatment effect. The key families for distinguishing among temperatures in descending order are Crocinitomicaceae, Nocardioidaceae, Azospirillaceae, and Moraxellaceae (see Fig. 5a and Supplemental Table S8). Only a few families help distinguish between populations, the most prominent of which is Rhodospirillaceae (Fig. 5b). In Fig. 6, the 19 families exhibiting a significant log2-fold change in relative abundance between low and high temperature are plotted (for complete results of this analysis among all pairs of temperature treatments, see Supplemental Table S7).

In addition to the main effects we found, we also observed an effect of extraction date on Bray-Curtis dissimilarity (*df* = 26, *F* = 2.7, *p* = 0.001), unweighted UniFrac (*df* = 26, *F* = 2.4, *p* = 0.001), and weighted UniFrac distances (*df* = 26, *F* = 2.91, *p* = 0.001), as was reported previously by Sullam et al. [44]. The microbiomes of samples from the source populations of each genotype prior to (“pre-”) and after (“post-”) the experimental period were compared to determine if there was a shift in the microbiota across genotypes during the course of the experiment (Supplemental Table S9). As detailed in the methods, these animals were not maintained under stringent sterile conditions, but instead reflect the baseline microbiome of lab-reared *D. magna* used as a source for experimental samples. Microbiomes from samples collected post-experiment differed from pre-experiment samples for Bray-Curtis dissimilarity (*df* = 1, *F* = 37.86, *p* = 0.001), unweighted UniFrac (*df* = 1, *F* = 30.19, *p* = 0.001), and weighted UniFrac (*df* = 1, *F* = 40.07, *p* = 0.001) distances. Data failed the homogeneity of group dispersion test for unweighted UniFrac (*df* = 1, *F* = 31.01, *p* = 0.001) and weighted UniFrac (*df* = 1, *F* = 5.55, *p* = 0.014) distances, suggesting the significance detected may be due to heteroscedasticity of data. However, NMDS plots show separation by pre- and post-experiment for all three metrics (Supplemental Fig. S1A-C), indicating that microbiomes of lab-reared populations used to source the experimental clonal lines changed in composition and structure over the duration of the experimental period. We also calculated and plotted the average relative abundance of the top 15 families (Supplemental Fig. S2) pooled for samples collected pre- and post-experiment. Notably, Acetobacterales-Incertae-Sedia and Moraxellaceae were not major components of the microbiota of stock populations prior to the start of the experiment but were relatively abundant in animals harvested from those same stock populations 6 months later, after the experimental period had ended.

To identify OTUs present in the kit, we performed extractions on just kit reagents and identified the top 20 OTUs present (which accounted for greater than 85% of the reads for these samples; Supplemental Table S10). We compared these OTUs to those detected in experimental samples (Supplemental Table S6). Five of the top 20 most abundant OTUs found in the kit reagents were also present in experimental samples, indicating a minor yet detectable fingerprint of kit contamination on the microbiome. Importantly, none of these OTUs impacted interpretations of significant families affecting characterizations of microbiomes (Fig. 5). In addition, we identified the top 20 OTUs present in PCR reagents and autoclaved ADaM (which accounted for greater than 85% of the reads for these samples; Supplemental Table S10). Four of the top 20 most abundant OTUs found in these samples were also present in experimental samples. One of the contaminant OTUs belonged to the family Moraxellaceae, one of the families significantly affecting microbiome composition (Fig. 5), and thus may have over-represented the significance of this family. Taken together, these findings further emphasize the need to identify contaminant sequences through comprehensive sequencing of negative controls.

## Discussion

Like many other eukaryotes, *Daphnia* harbor an abundant community of microbes, externally and internally [31]. While the *Daphnia* gut microbiome is dominated by a few community members [18], the whole-body microbiome is known to consist of many more taxa [31]. Previous studies have shown that the *Daphnia* microbiome impacts host fitness in multiple ways (e.g., affecting lifespan, fecundity, and body size [41]), and there is evidence that specific community members may have disproportionate effects [29]. Here, we aimed to uncover whether the *Daphnia* microbiome exhibits temperature-dependent effects or varies among populations-of-origin in lab-reared clones raised under standardized conditions many years post-collection. Overall, our findings suggest an important role for both temperature and population-of-origin in determining the microbiome in this species, as well as an unanticipated effect of extraction date, suggesting expected and unexpected abiotic factors influence microbial communities in a laboratory-based study system.

The largest effect that we observe on the microbiome of *D. magna* is temperature (Table 1; Figs. 2 and 3). This result corroborates a previous study reporting temperature effects on measures of Bray-Curtis dissimilarity and unweighted UniFrac distance, but now shows those effects can be observed over a much smaller range of temperatures (i.e., Low [16 °C], Medium [18 °C], and High [20 °C] in our study, versus Low [20 °C] and High [28 °C] in Sullam et al. [44]), and in an additional beta diversity metric (weighted UniFrac distance; Table 2). These results suggest small scale changes in environmental temperature significantly impact microbiomes. The temperature gradient used in the study presented herein represents temperature changes of 2 °C, reasonably representing environmental temperature shifts due to climate change in the near future. These findings complement those of Sullam et al. [44] and provide additional information on microbiome plasticity in response to temperature.

Due to differences in sampling design between the two studies, the effect of “host clonal line” (from Sullam et al. [44]) versus the effect of population-of-origin and genotype cannot be directly compared. Our study sampled three genotypes from three populations; Sullam et al. [44] sampled one genotype per population from 18 populations. While not identical, we interpret an effect of population in our study to be similar to an effect of “host clonal line” in Sullam et al. [44]. We detect a population-of-origin effect using Bray-Curtis dissimilarity and report an additional effect using weighted UniFrac distances. Similar to results reported in Sullam et al. [44], we observe no temperature or population-of-origin effects on Simpson or Shannon diversity indices, although pairwise differences between some genotypes are observed (Fig. 4c). We originally hypothesized a difference in response to temperature based on population-of-origin, given the differing climatic regimes *Daphnia* face in Finland, Germany, and Israel, but no interaction effect was observed (Table 1), in contrast to Sullam et al. [44].

Because OTUs or species-rank names may not be comparable across studies due to differing methodologies and databases, we compared community compositions at the rank of family. Among the most relatively abundant families reported in our study, two (Flavobacteriaceae and Burkholderiaceae/Comamonadaceae) were also highly abundant in Sullam et al. [44]. Although there is a temperature effect detected in the overall analysis discussed above, there are no temperature effects observed for the most relatively abundant taxa after Bonferroni correction using parametric tests (Table 3), although non-parametric Kruskal-Wallis tests show a significant effect of temperature for Burkholderiaceae (Supplemental Table S4F). This result is not surprising, given that the most abundant taxa might be expected to be most resilient to environmental shifts.

In terms of presence/absence, the key families for correct classification of samples based on temperature treatment were not the same as the most abundant taxa (Fig. 5), nor were the families exhibiting the greatest log2-fold changes the most abundant (Fig. 6). It is possible that changes in only rare taxa mean there is little impact of temperature on function. However, an important take-home message from this study, and perhaps more generally, is that the most abundant taxa and the key changing taxa are not necessarily the same, making the sensitivity levels of different studies extremely important, because the ability to detect less abundant taxa depends largely on the depth of sequencing per sample. This is especially important for follow-up studies that might focus on the functional biology of the microbiome in an effort to understand the roles various taxa might play in determining the health or physiology of their hosts. While the potential effect of rare taxa on host function may be thought to be less than the most abundant taxa, these results suggest that variation among low abundance taxa exists and should not be ignored. Likewise, although we were able to reproduce many of the large-scale observed patterns from Sullam et al. [44], the use of different databases prevents us from determining if the observed effects on specific bacterial OTUs are also repeatable. This should be of considerable interest for future inquiries into the reproducibility of microbiome studies.

Because we found an effect of extraction date (Table 1) like [44], we wanted to investigate the potential role of colonization or contamination during the experiment. To test whether the duration of the experimental period affected the likelihood of seeing an extraction date effect, we performed temperature-specific ANOVAs with the hypothesis that low-temperature treatments would exhibit the strongest effect of extraction date, given that those animals were in the lab the longest and thus most likely to experience spurious, time-based colonization events. In contrast to this expectation, the low-temperature treatment exhibited the least effect of extraction date (Supplemental Table S4). Our samples collected pre-/post-experimental period differed in their microbiome (Supplemental Fig. S1, Supplemental Table S9). This may be important, as it is often presumed that lab-reared populations maintain a stable microbiome once acclimated to rearing conditions, but this indicates a change over a 6 month period. Another potential explanation was kit contamination (see Salter et al. [34]), and we identified abundant OTUs detected in extraction kit reagents and compared them to OTUs identified in our experimental samples. None of these putatively contaminant OTUs influenced our major findings, though some were detected in our samples. However, identification of contaminant OTUs in the other negative controls sequenced (PCR reagents and autoclaved ADaM) revealed one putative contaminant OTU belonging to a family that we found to be significant in our analysis. These results emphasize the importance of controlling for all possible sources of non-experimental variation through comprehensive sequencing of negative control samples throughout the course of a microbiome study.

## Conclusions

While our study and that of Sullam et al. [44] differed in a number of significant ways (e.g., sampling strategy, temperatures tested, xenic vs. axenic laboratory conditions), similar major effects were observed in both studies (i.e., temperature and population-of-origin effects). The reproducibility of these results across experiments and laboratories majorly increases our confidence in the robustness of our main findings. Furthermore, given that effects reported in Sullam et al. [44] are corroborated by our study (with a narrower range of temperatures, fewer populations, but more genotypes per population), these data suggest that variation in the *Daphnia* microbiome might be even more sensitive to changes in such factors than previously thought. Ultimately, reproducibility is essential for identifying major causes of change to the microbiome and delimiting the thresholds of those effects. Without reproducing studies, the danger of reporting laboratory-specific effects cannot be eliminated. Recent efforts, such as the Microbiome Quality Control Project [38], have helped identify laboratory-specific factors influencing microbiome studies of humans [39], but rigorous standards have not been developed and may not be prioritized for other systems.

Given the immense importance of the *Daphnia* system in ecology and environmental science as a bioindicator species, this is a crucial study system for investigating shifts in the microbiome. Our data provide evidence such shifts occur based on temperature (Tables 1 and 2, and Figs. 2 and 3) and population-of-origin (Table 1, Fig. 3a, c). Future studies should investigate the microbiome differences among *Daphnia* isolates immediately post-collection from across a latitudinal gradient and look at the role of temperature fluctuations on microbiome content and stability. Other studies of microbiome response in near-future temperature regimes have shown a wide range of patterns (from no variation to major variation affecting microbiome functionality), suggesting the need for more research [2, 32]. Further investigation, with reproducible results, looking into whether shifts in microbiota are common across temperature gradients (in nature and in the laboratory) will clarify our understanding of how such factors might shape microbiome variation as climates change.

## Electronic supplementary material

Supplemental Figure S1 Non-metric multidimensional scaling (NMDS) plots based on (A) Bray-Curtis dissimilarity, (B) unweighted UniFrac distance, and (C) weighted UniFrac distance for samples collected pre- and post- the experiment was conducted. (PDF 6 kb)

A (PDF 6 kb)

Supplemental Figure S2 Average relative abundance of the top 15 families found in pooled samples originally collected from Israel (Top) and Germany (Bottom) based on extractions performed before (“pre-”) and after (“post-”) the experimental period. (PDF 17 kb)

B (PDF 17 kb)

C (PDF 17 kb)

ESM 1 (RMD 32 kb)

ESM 2 Supplemental Table S1 Sample locales. Supplemental Table S2. Sample Metadata. Metadata for individual samples and sequencing read counts throughout the DADA2 filtering pipeline. Supplemental Table S3. Primer + Adaptor Seqs. Supplemental Table S4. ANOVA Tables. Raw ANOVA tables output from PERMANOVA (summarized in Table 1), ANOVA (summarized in Table 2), and post hoc pairwise PERMANOVA (summarized in Table 3). Supplemental Table S5. Alpha Diversity Metrics. Alpha diversity statistics for observed richness, Simpson and Shannon metrics. Supplemental Table S6. Relative Abundance >1%. Relative abundances for experimental samples grouped at the family rank at a 1% threshold. Supplemental Table S7. DESeq2 Data Table. Log2-fold differential abundance in temperatures across families (used to make Fig. 6). Supplemental Table S8. Mean Classification Accuracy Data (used to make Fig. 5). Supplemental Table S9. Pre- and Post- Experiment ANOVA tables. Supplemental Table S10. Kit Contaminant OTUs. Top 20 relative abundance OTUs detected in DNA extraction kits, PCR reagents, and autoclaved ADaM. (XLS 899 kb)
