# Supplementary figures and images for: Variation in the Microbiota Associated with Daphnia magna Across Genotypes, Populations, and Temperature
Source: Microb Ecol. 2019 Aug 3;79(3):731–42. doi: 10.1007/s00248-019-01412-9 (PMC7176607; doi:10.1007/s00248-019-01412-9)

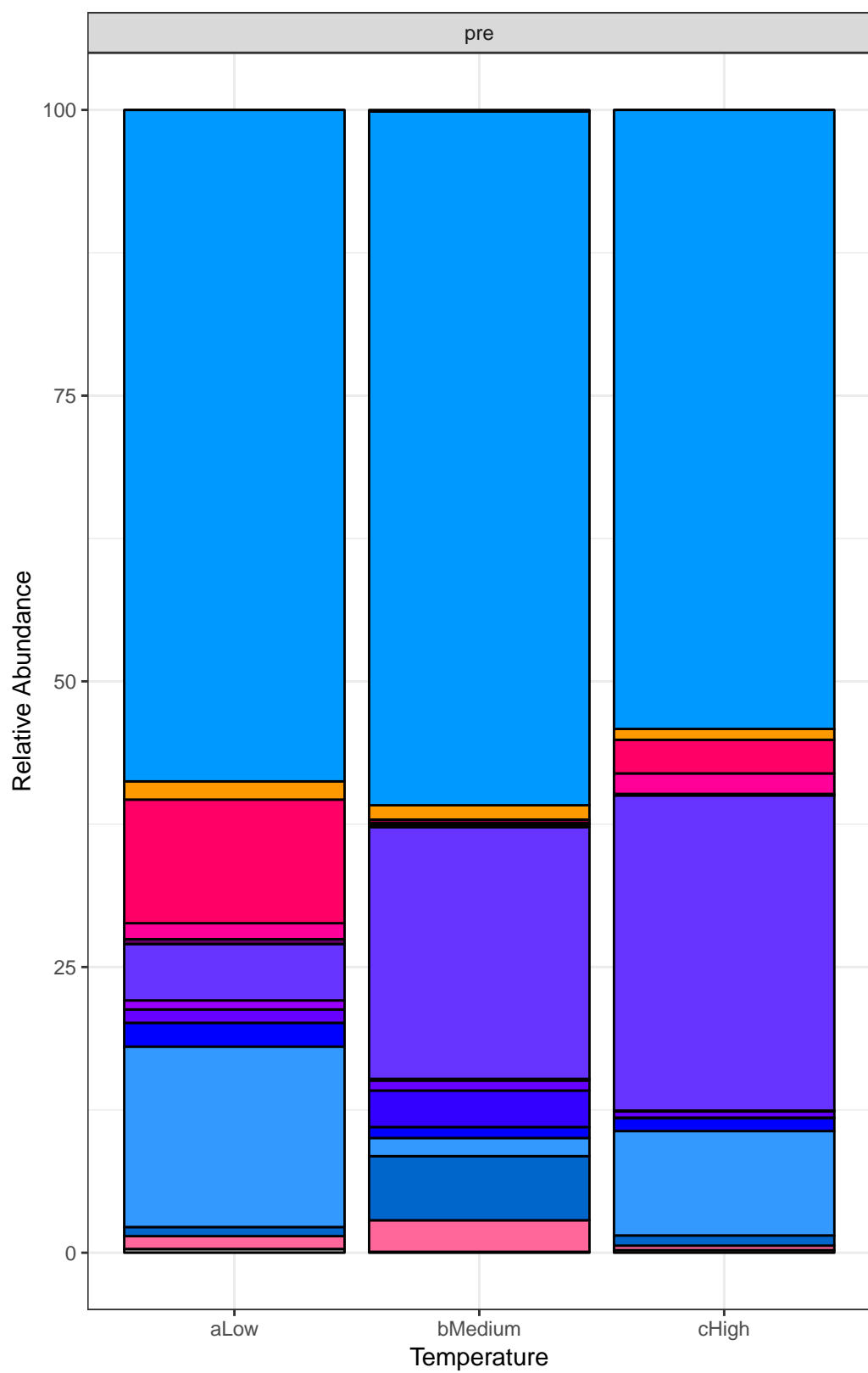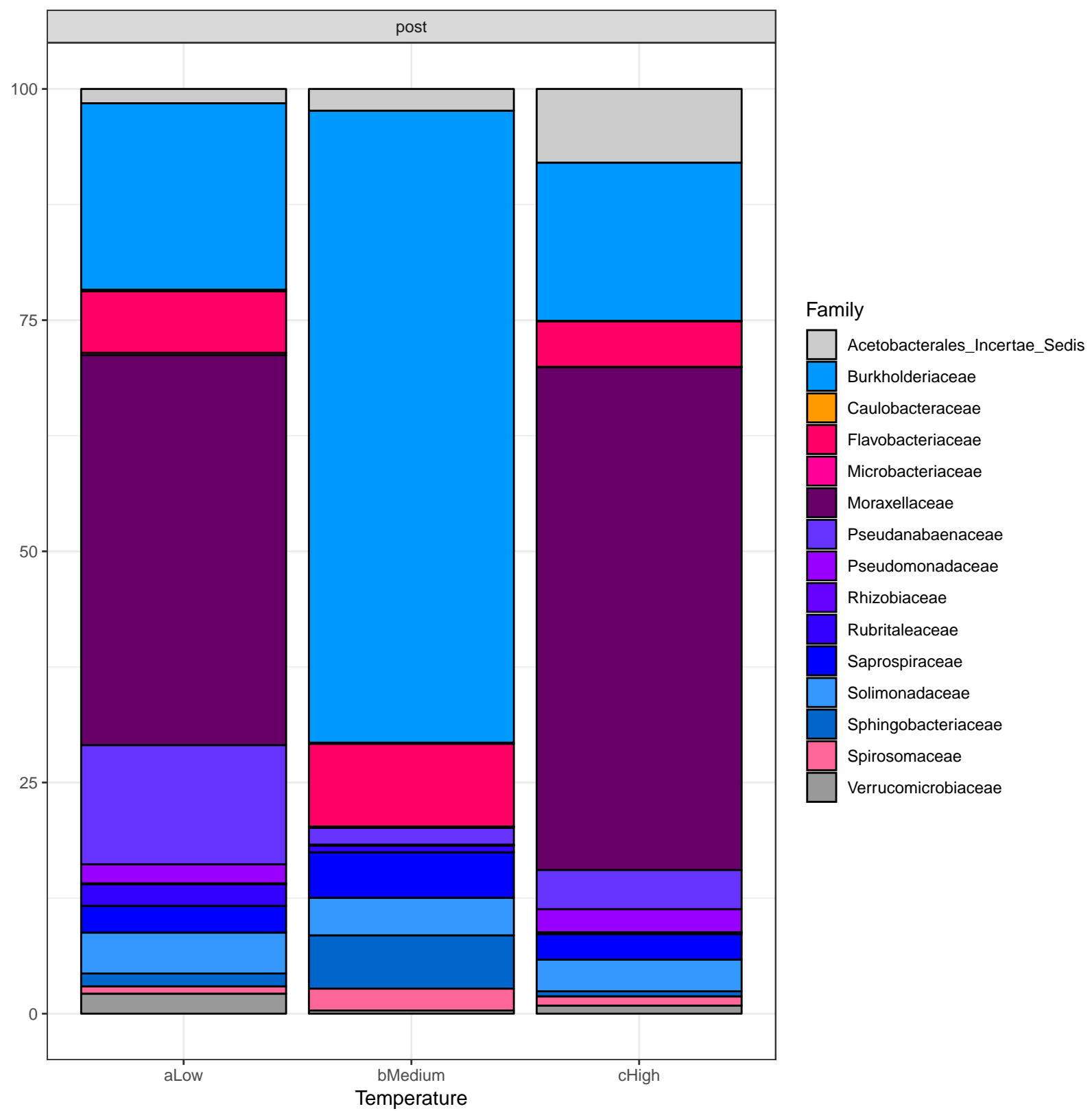

Supplement: Supplementary file 1 — Non-metric multidimensional scaling (NMDS) plots based on (A) Bray-Curtis dissimilarity, (B) unweighted UniFrac distance, and (C) weighted UniFrac distance for samples collected pre- and post- the experiment was conducted. (PDF 6 kb) [file 248_2019_1412_MOESM1_ESM.pdf]

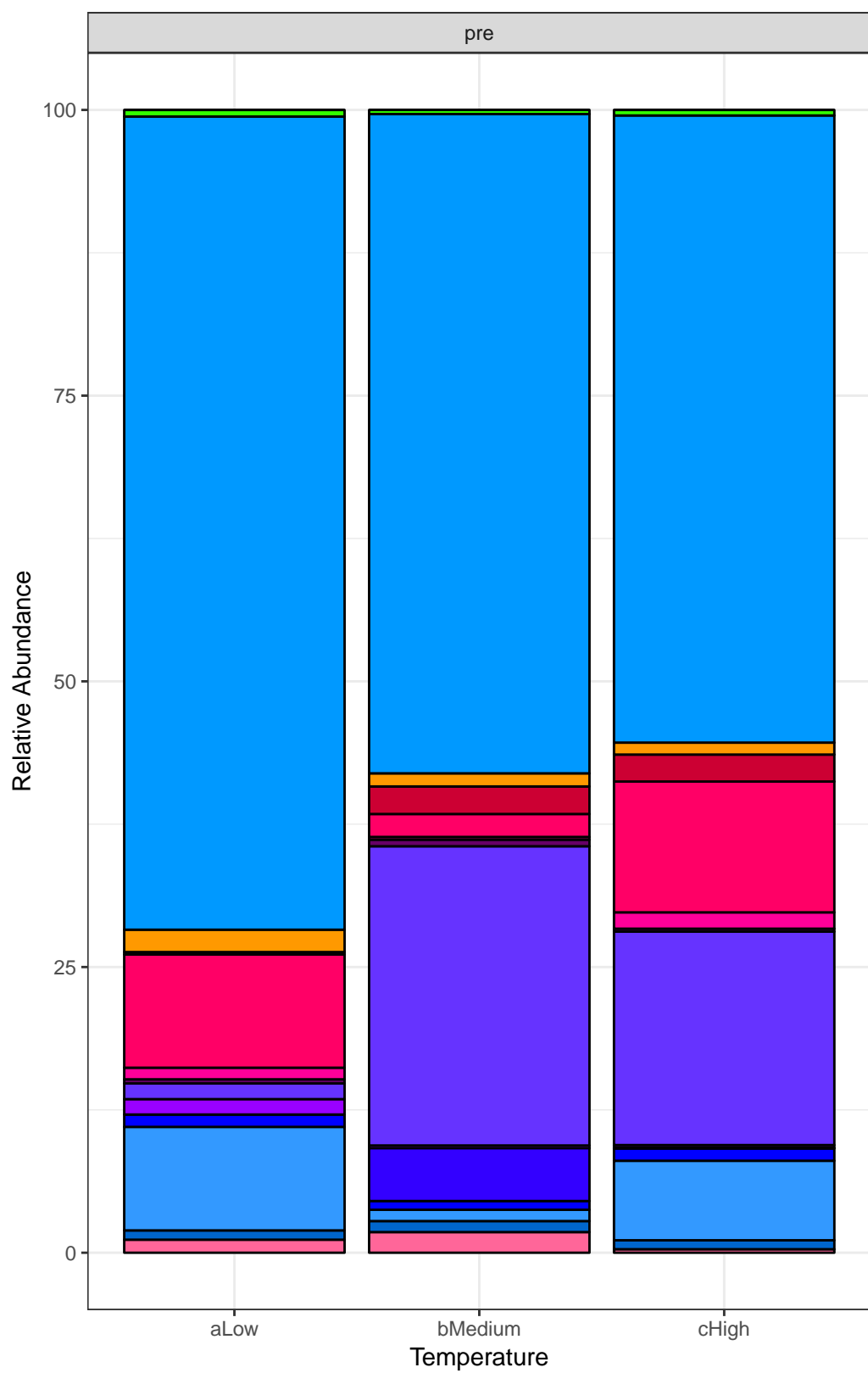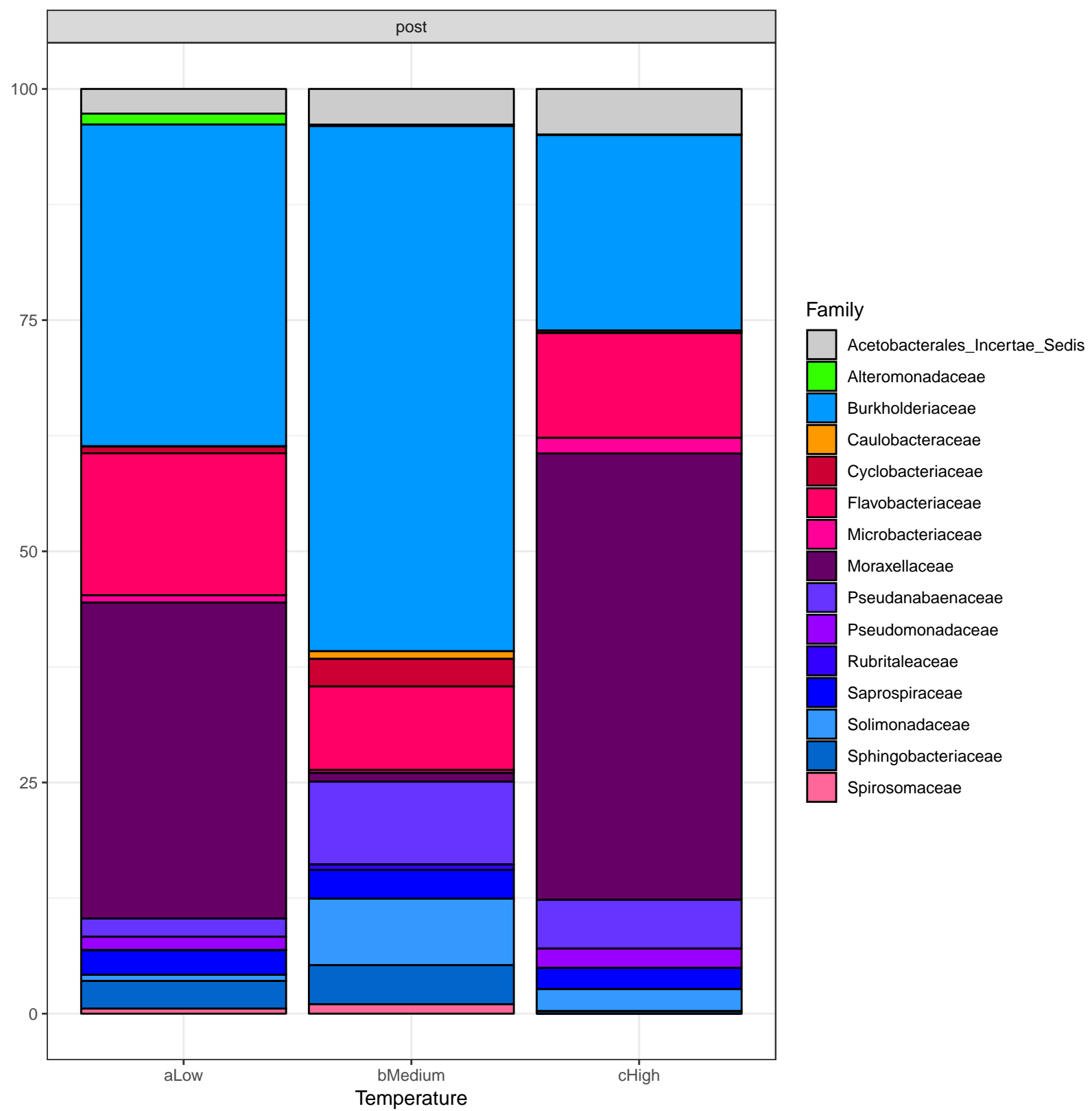

Supplement: Supplementary file 2 — A (PDF 6 kb) [file 248_2019_1412_MOESM2_ESM.pdf]

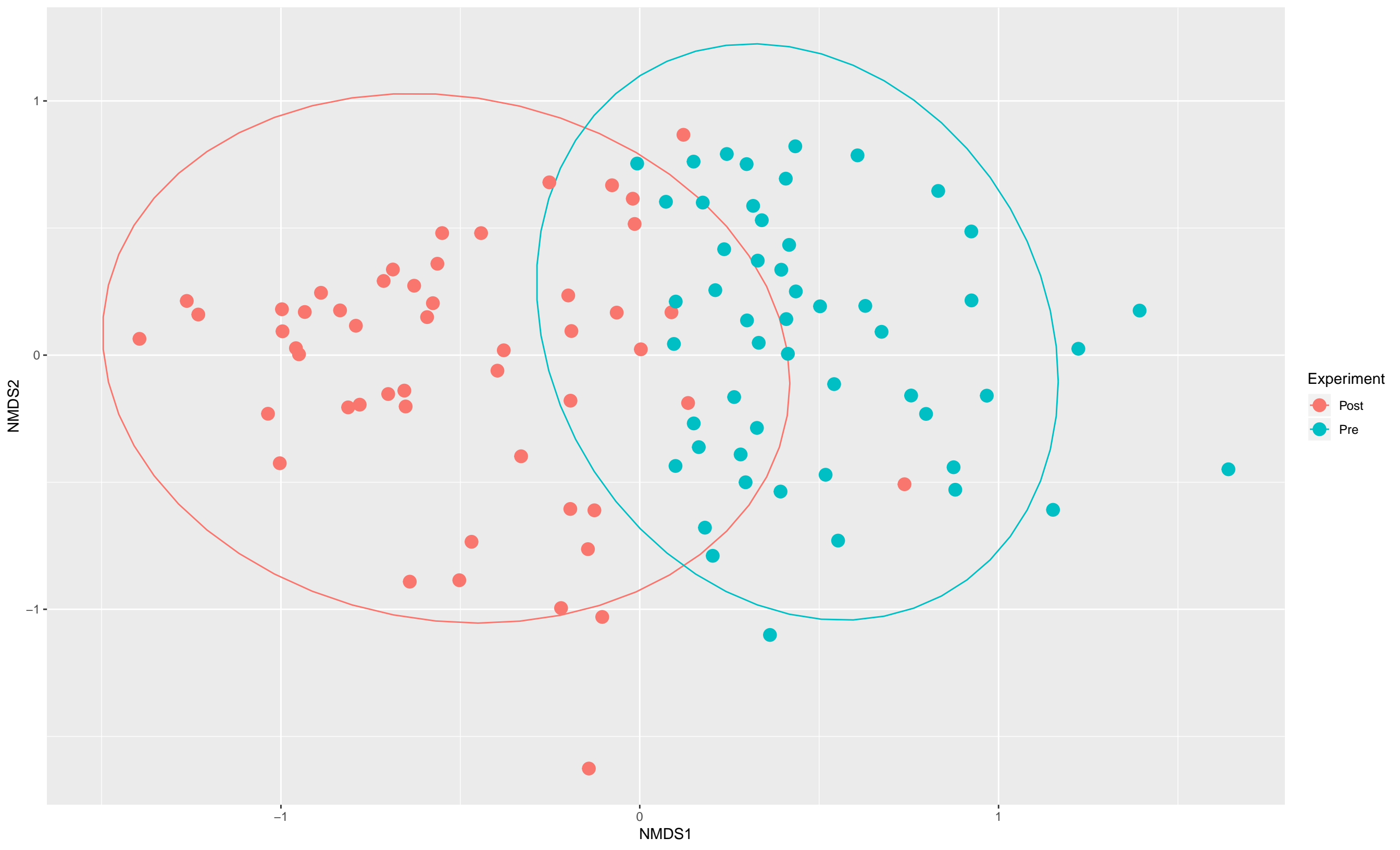

Supplement: Supplementary file 3 — Average relative abundance of the top 15 families found in pooled samples originally collected from Israel (Top) and Germany (Bottom) based on extractions performed before (“pre-”) and after (“post-”) the experimental period. (PDF 17 kb) [file 248_2019_1412_MOESM3_ESM.pdf]

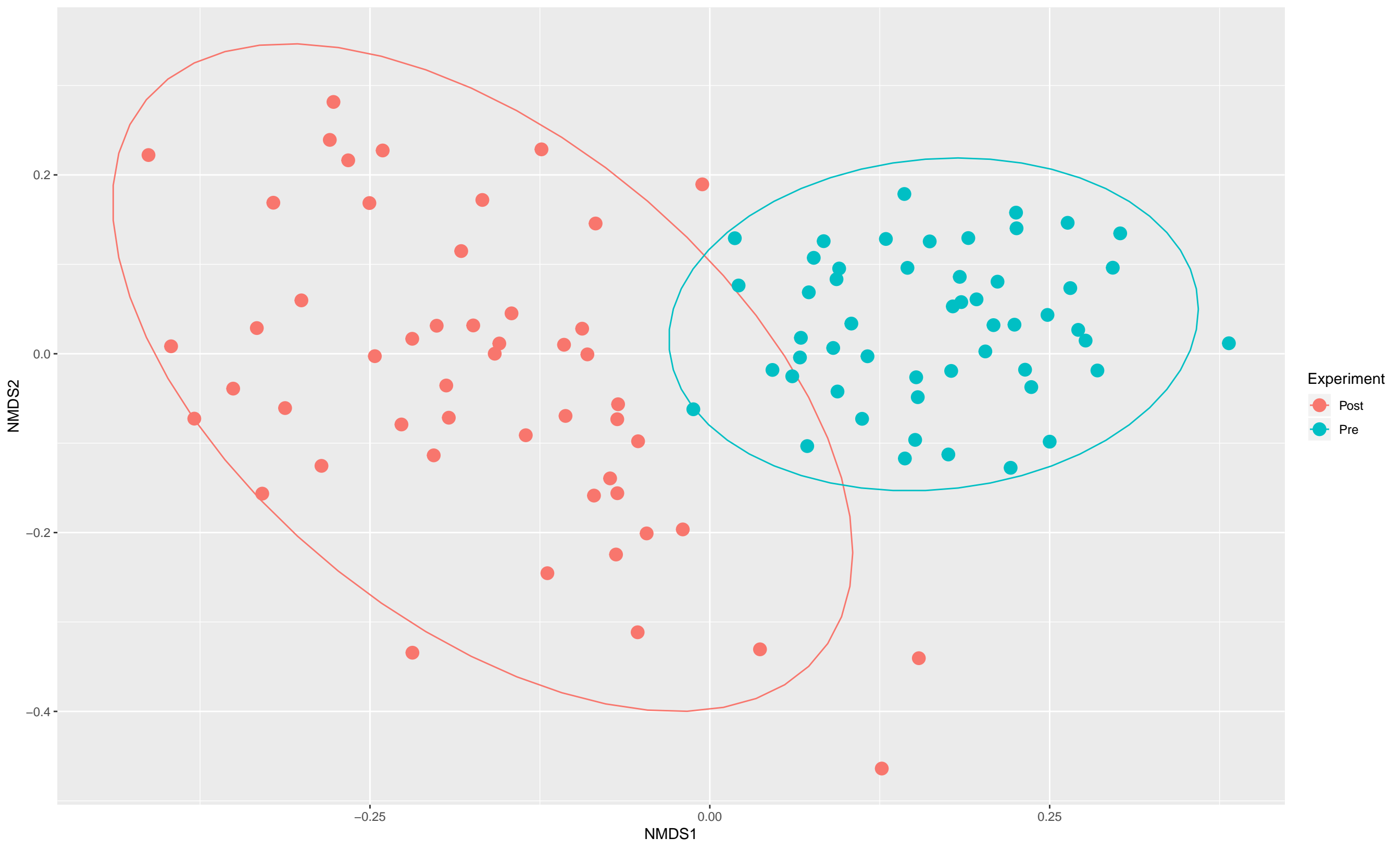

Supplement: Supplementary file 4 — B (PDF 17 kb) [file 248_2019_1412_MOESM4_ESM.pdf]

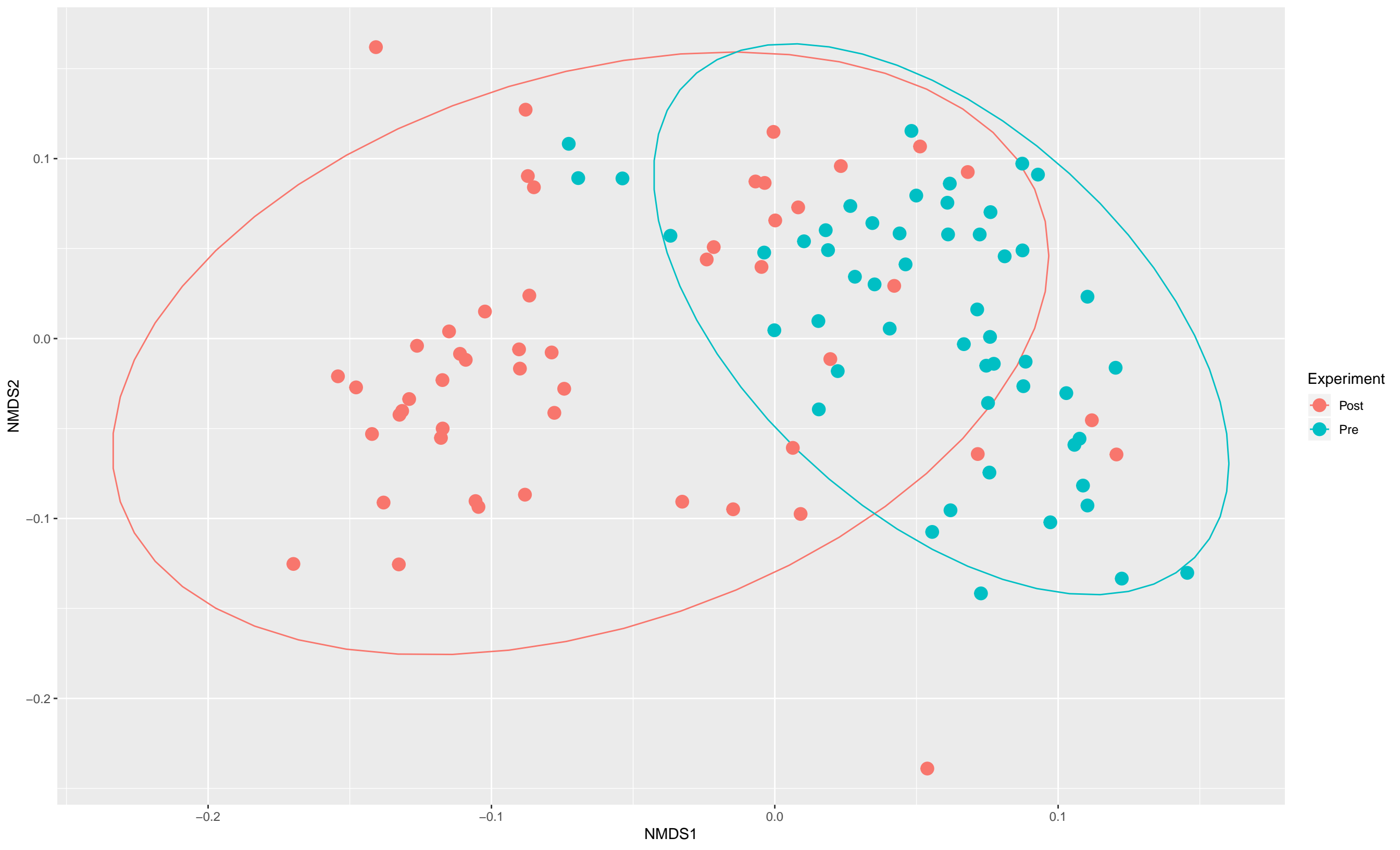

Supplement: Supplementary file 5 — C (PDF 17 kb) [file 248_2019_1412_MOESM5_ESM.pdf]
